# Cytotoxicity and Oxidative Stress Effects of Indene on Coelomocytes of Earthworm (*Eisenia foetida*): Combined Analysis at Cellular and Molecular Levels

**DOI:** 10.3390/toxics11020136

**Published:** 2023-01-30

**Authors:** Chengqian Huo, Qiang Zhao, Rutao Liu, Xiangxiang Li, Falin He, Mingyang Jing, Jingqiang Wan, Wansong Zong

**Affiliations:** 1 School of Environmental Science and Engineering, Shandong University, China-America CRC for Environment & Health, 72# Jimo Binhai Road, Qingdao 266237, China; 2 Shandong Provincial Eco-Environment Monitoring Center, 3377 Jingshi Dong Lu, Jinan 250100, China; 3 College of Population, Resources and Environment, Shandong Normal University, 88# East Wenhua Road, Jinan 250014, China

**Keywords:** indene, superoxide dismutase, coelomocytes, oxidative stress, interaction mechanisms

## Abstract

Indene (IND) is a kind of important aromatic hydrocarbon that is extracted from coal tar and has important applications in industry and biology. In the process of production and utilization, it is easy to enter the soil and produce toxic effects on the soil or organisms. The earthworm is an important organism in the soil. The toxicity of indene on earthworm coelomocytes is rarely studied, and the oxidative stress effects of IND on earthworm coelomocytes remain unclear. In this study, coelomocytes from earthworms and antioxidant enzymes were selected as the research targets. In addition, IND caused oxidative stress, and its related toxic effects and mechanisms were systematically studied and evaluated at the cellular and molecular levels. The results showed that IND destroyed the redox balance in earthworm coelomocytes, and the large accumulation of reactive oxygen species (ROS) significantly inhibited the activities of the antioxidant system, including superoxide dismutase (SOD), catalase (CAT), and glutathione (GSH), and caused lipid peroxidation and membrane permeability changes, resulting in a decrease in cell viability to 74.5% of the control group. At the molecular level, IND was bound to SOD by the arene-H bond, and the binding constant was 4.95 × 10^3^. IND changed the secondary structure of the SOD and led to a loosening of the structure of the SOD peptide chain. Meanwhile, IND caused SOD fluorescence sensitization, and molecular simulation showed that IND was mainly bound to the junction of SOD subunits. We hypothesized that the changes in SOD structure led to the increase in SOD activity. This research can provide a scientific basis for IND toxicity evaluation.

## 1. Introduction

Through years of development, the domestic coal tar industry has made great progress in industrial scale, processing depth, product diversification, and so on. Accordingly, the output of coal tar has increased year over year. Coal tar is an organic mixture dominated by aromatic hydrocarbons, containing more than 10,000 compounds, and about 200 kinds can be extracted [1]. It is widely used in synthetic plastics, pesticides, medicine, high temperature resistant raw materials, the national defense industry, and other fields, among which some compounds cannot be produced and must be replaced by the petroleum processing industry [2]. With the continuous development of the economy and the increasing requirement for environmental protection, soil polluted by coal tar has become an urgent problem to be solved.

Cameron et al. noted that exposure to high concentrations of some samples of heavy coal tar naphtha produced toxic effects in mice [3]. In high-temperature tar, the content of indene (IND) is 0.25–0.3%, which mainly exists in coal tar and crude benzene fractions with a boiling point of 168–175 °C. The IND has important applications in industry and biology [4,5]. It can be used to produce IND-magolon resin and styrene-IND resin [6,7]. High purity IND is also an important comonomer, which can be used to change the surface properties of polymers [8]. However, as an environmental pollutant, its target organs are the eyes, skin, respiratory system, liver, kidney, and spleen, which can stimulate the skin and mucous membranes, and cause damage to the liver, spleen, kidney, and lung by high concentrations of contact (NIOSH, 2016). In rabbits, the IND can be converted into cis-1,2-dihydroxyindane and trans-1,2-dihydroxyindane [9]. Some studies have found that kidney injury, liver injury, and occasionally spleen injury were found in rats after 6 and 7 h exposure to a concentration of 800–900 PPM of IND steam. Some rats developed severe hemorrhagic liver necrosis, with renal histological changes including focal necrosis similar to small infarcts [10].

The toxic effects of exogenous pollutants are often evaluated by an oxidative stress index in experiments [11,12]. When the redox balance in the intracellular environment is destroyed, reactive oxygen species (ROS) accumulate in large quantities and the intracellular environment tends to oxidize, thus inducing oxidative stress. Antioxidant enzymes such as superoxide dismutase (SOD), catalase (CAT), and glutathione peroxidase (GSH-Px) form the enzymatic antioxidant system, which is generally regarded as the first line of defense in the body [13]. In the anti-oxidative stress defense system, SOD as the first line of defense can catalyze superoxide anion free radicals (O_2_^−^) to generate hydrogen peroxide (H_2_O_2_) [14]. H_2_O_2_ generates O_2_ and H_2_O under the catalysis of CAT, which protects cells from H_2_O_2_ damage [15]. Studies have reported that the blood cholinesterase activity increased and catalase activity was inhibited in rats after continuous exposure to 3 mg/m^3^ IND smoke from plastic floor tiles for 105 days [16].

The earthworm is one of the most important organisms in soil, is widely distributed in soil, and is sensitive to exogenous pollutants in soil [17,18]. Earthworms have been used in many scientific studies to evaluate the toxicity of soil pollutants [19]. *Eisenia foetida* was used as a model organism for standard toxicology tests by the Organization for International Economic Cooperation and Development (OECD) [19,20].

There have been a lot of studies evaluating the toxic effects and mechanisms of pollutants at the molecular and cellular levels, but it is not clear whether the same toxic effects induced by the same pollutant at the molecular and cellular levels are correlated. Herein, indene was selected as a pollutant, the coelomocytes of earthworms as target cells, and SOD as a target protein to study whether there is a close correlation between indene and oxidative stress induced by indene at the cellular and molecular levels and to clarify the mechanism of oxidative stress induced by indene at the molecular and cellular levels. The molecular mechanisms of interactions between IND and key antioxidant enzymes (SOD) were studied by using multispectral methods, molecular docking (MOE), and isothermal calorimetry (ITC). This research provides a strategy to investigate the response of biomarkers to pollutant exposure at the cellular and molecular levels. In addition, this research can provide a reference for minimizing the toxic effects of IND.

## 2. Materials and Methods

### 2.1. Chemicals and Reagents

IND (analytically pure grade; 98%) was obtained from Macklin, Shanghai, China. SOD was obtained from Solarbio (Beijing, China). RPMI 1640, streptomycin/penicillin, and fetal bovine serum were all obtained from Thermo Scientific Co., Ltd. (Waltham, MA, USA). Na_2_HPO_4_·12H_2_O and NaH_2_PO_4_·2H_2_O were extracted by Sinopharm Chemical Reagent Co., Ltd (Shanghai, China). All the chemicals were of analytical grade. All experiments used ultrapure H_2_O (18.25 MΩ cm) from the Millipore Milli-Q Water Purification System (Billerica, MA, USA). All the kits were purchased from Nanjing Jiancheng Institute of Biological Engineering (Nanjing, China).

### 2.2. Test Organisms

Adult earthworms (3–5 months old) were selected as the subjects and purchased from Wangjun Earthworm Breeding Company, Jurong City, Jiangsu Province. Earthworms were cultured in a climate incubator under constant temperature (20 °C) and humidity in the artificial soil (70% quartz sand, 20% kaolin, and 10% peat soil) for a week.

### 2.3. Intracellular Oxidative Stress Analysis

#### 2.3.1. Extraction of Earthworm Coelomocytes and IND Exposure Experiment

After a week of domesticating earthworms, we randomly selected a set number of earthworms as we needed for the experiment, washed them with ultrapure water, and used a hairless absorbent paper to remove the excess water from the surface of the earthworms. Please refer to the Supplementary Materials for the specific experimental process of extracting earthworm coelomocytes. On the basis of pre-experiments, we incubated earthworm coelomocytes with IND (0, 0.1, 0.5, 1, 5, and 15 mg/L) at 25 °C for 24 h, of which the 0 mg/L experimental group is the blank control group.

#### 2.3.2. Cell Viability Assay

We added 10 μL CCK-8 chromogen to each well with a multi-channel pipette (be careful not to form bubbles) after incubation at 20 °C in CO_2_ free for 24 h. After incubation at room temperature for 2 h, the A_450_ (absorption value at 450 nm) was measured with a microplate meter, and the cell viability was calculated according to the following formula:

(1) $$cell\;vitality\%=\frac{OD_{3}-OD_{2}}{OD_{1}-OD_{0}}\times 100$$

where *OD*_3_ refers to the absorbance of the experimental group, *OD*_2_ to the experimental reference group, *OD*_1_ to the blank group, and *OD*_0_ to the blank reference group, respectively.

Finally, the calculated results were based on the 100% cell viability of the blank group.

#### 2.3.3. ROS Determination

The content of ROS in the organism was determined by comparing fluorescence intensities. The cultured coelomocytes were centrifuged (3500 RPM, 5 min) and washed once with normal saline under the same centrifugation conditions. Then 100 μL normal saline containing 1 μM DCFH-DA probe was added, and the coelomocytes were blown and washed into the probe suspension. The coelomocytes were incubated at 20 °C for 40 min, and the fluorescence intensity was measured with a luciferase plate analyzer.

#### 2.3.4. Antioxidant Enzyme Activity and Malondialdehyde (MDA) Level Determination

After being cultured for 24 h, the earthworm coelomocyte fluid was centrifuged (3500 RPM, 5 min). The supernatant was poured out, and washed twice with normal saline under the same centrifugation conditions. The coelomocytes were treated with 20% amplitude ultrasound and centrifugated at 5000 RPM for 5 min at 4 °C. Then the supernatant was collected to determine the total SOD activity, CAT activity, GSH content, and MDA level. According to the kit instructions (Nanjing Jiancheng Biological Engineering Institute, Nanjing, China), the total SOD activity, GSH content, and MDA level were calculated by using a UV-2600 spectrophotometer (Shimadzu, Kyoto, Japan) to measure A_550_, A_405_ and A_532_, respectively.

CAT activity was evaluated by measuring the absorbance change rate of H_2_O_2_ at 240 nm [21]. 300 μL supernatant was quickly mixed with 2.7 mL hydrogen peroxide (10 mM, diluted with normal saline), and then A_240_ was recorded by using a UV-2600 spectrophotometer (Shimadzu, Kyoto, Japan). Using ultrapure water as a reference, the changes in A_240_ were measured every 30 s within 3 min. After the measurement, the measured data were drawn as a scatter plot, and linear fitting was performed. The slope of the line was the change rate of H_2_O_2_ absorbance (the X axis was the measurement time, and the Y axis was the value of A_240_ every 30 s).

#### 2.3.5. Lactate Dehydrogenase (LDH) Activity Determination

The activity of pyruvate was detected by the principle that LDH can catalyze lactic acid to generate pyruvate. Pyruvate can react with 2,4-dinitrophenylhydrazine to generate pyruvate dinitrophenylhydrazone, which has a large absorption at 450 nm.

After the coelomocytes were exposed to IND for 24 h, they were centrifuged at 1500 RPM at 4 °C for 5 min. The supernatant was collected, and LDH activity in the supernatant was detected. A_450_ of each well was measured using a microplate reader according to the kit instructions.

#### 2.3.6. Na^+^ K^+^ ATPase Activity Determination

Using the principle that ATPase can decompose ATP to generate adenosine diphosphate (ADP) and inorganic phosphorus, the activity of ATPase can be determined by detecting the amount of inorganic phosphorus. After being cultured for 24 h, the coelomocytes were centrifuged (3500 RPM for 5 min), the supernatant was discarded, and the underlying coelomocytes were left behind. 0.2–0.3 mL of normal saline was added to each tube to prepare 10^7^/mL of suspension, and then ultrasonic crushing was performed. The broken cell suspension was taken for the determination of Na^+^ K^+^ ATPase activity. After adding reagents according to the kit, the mixture was allowed to stand at room temperature for 5 min, and the absorbance value at 636 nm of each tube was measured.

#### 2.3.7. Total Protein (TP) Determination

The cultured cells were centrifuged, blown, and broken according to the above method, then centrifuged again at 4 °C (5000 RPM, 5 min), and stood at room temperature for 5 min. 50 μL supernatant was taken as the sample to be tested. The absorbance of each tube at 595 nm was measured according to the kit instructions.

ROS level, total SOD activity, CAT activity, GSH content, MDA level, LDH activity, and Na^+^ K^+^ ATpase activity were determined together with total protein content, and the final results of each index should be expressed on the basis of the same TP amount.

### 2.4. Analysis of Functional and Structural Changes of SOD

#### 2.4.1. Reaction System Configuration

Reagent configuration: phosphate buffered (PB, Na_2_HPO_4_-NaH_2_PO_4_, 0.2 M) with a pH of 7.4, SOD, and IND reserve solutions with certain concentrations. Experimental group: 1mL buffer solution, different volumes of IND solution, and 1mL SOD solution were successively added into a 10 mL centrifuge tube. The samples were diluted to a constant volume of 10 mL and reacted for 1 h at 25 °C.

#### 2.4.2. SOD Activity Assay

After 1 h constant temperature reactions in the molecular reaction system, three groups were set up in parallel in the experiment. Take 100 μL of 10-fold dilution reaction solution as the sample to be tested. SOD activity was measured according to the kit instructions.

#### 2.4.3. Isothermal Calorimetric Titration (ITC)

ITC experiments were performed on a Malvern MicroCal PEAQITC (Malvern Instruments Ltd., Malvern, UK) microcalorimeter to investigate the thermodynamic variables consisting of the enthalpy (ΔH) change, binding affinity constant (K), entropy (ΔS) change, and the binding stoichiometry (N) [22]. Dissolve the SOD and IND in a buffer of the same concentration (excessive dilution heat due to buffer mismatch should be avoided) and filtrate through a 0.22 μm membrane. In the experimental group, the IND solution was absorbed into the titration needle, and the protein solution was placed in the reaction cell with no bubbles during the process. The titration parameters were set as follows: temperature: 25 °C, titrating needle speed: 1000 RPM, 2 min interval for each drop, a total of 14 drops, of which the first drop was a bad spot (test drop), the titrating volume was set as 0.4 μL, the others as 3 μL. The thermodynamic data of intermolecular binding can be quickly obtained by fitting the data after titration. Gibbs free energy change (ΔG) was determined with the equation:

ΔG = ΔH − TΔS = −RTlnK(2)

where R designates the gas constant and T represents the thermodynamic temperature.

#### 2.4.4. Fluorescence Spectrum Experiment

Fluorescence spectrum and synchronous fluorescence spectrum, excitation-emission-matrix (EEM) and resonant light scattering spectrum (RLS) were measured on a fluorescence spectrophotometer. Please refer to the Supplementary Materials for specific experimental parameters.

#### 2.4.5. Internal Rate Deduction Experiment

The internal filtration effect (IFE) was measured using a UV-Vis spectrophotometer with ultrapure water as a reference. The absorption values were measured in the wavelength range of 240–460 nm, the slit width was set at 2 nm, and the sampling interval was 0.2 nm. The fluorescence intensity was corrected by the following formula:

(3) $$F_{cor}=F_{obs}\times 10^{\frac{Aex+Aem}{2}}$$

where *F_cor_* and *F_obs_* refer to the fluorescence intensity after correction and the actual measurement, respectively. *Aex* and *Aem* refer to the absorbance of the experimental system at excitation and emission wavelengths, respectively.

#### 2.4.6. UV-Vis Absorption Spectra Experiment

The absorbance was measured by a UV-2600 spectrophotometer, ultrapure water was set as the baseline, and the reference was set as the reaction system with the corresponding series concentration without SOD (replacing SOD with ultrapure water of the same volume). The absorbance value was measured in the wavelength range of 190–500 nm, the width of the slit was set at 2 nm, and the sampling interval was 0.5 nm.

#### 2.4.7. Circular Dichroic Spectra Experiment

The circular dichroic spectra of the SOD molecular reaction system were determined by its dichroic spectra. We used a 1 mm optical diameter colorimeter to deduct the blank value of phosphate buffer solution. The sampling interval was set at 0.5 nm, and the measurement wavelength range was 190–250 nm.

#### 2.4.8. Molecular Docking Experiment

Molecular Operating Environment software (MOE, Chemical Computing Group Inc., Canada) was used for molecular docking. The molecular structure of SOD is provided by the RSCB Protein Data Bank platform (http://www.pdb.org/, accessed on 15 June 2021), and the PDB code is 2SOD. The molecular structure of IND is composed of the small molecule database ZINC (http://zinc.docking.org/ (accessed on 16 December 2022)) (ZINC ID 1699888). Before molecular butting, water molecules and heterochains of macromolecule proteins are removed, and then hydrogenation operations are carried out to minimize the energy. The Sitefinder module was used to find the active pockets of macromolecular proteins, the Dock panel was used to calculate the most appropriate binding sites and the exposure of nearby amino acids, and the Surfaces and Maps module was used to display and beautify the local binding map.

### 2.5. Statistical Analysis

Each cellular indicator was independently repeated three times. The experimental data were compared between the blank control group and the experimental group using Dunnett’s method in one-way analysis of variance (ANOVA) in the statistical software GraphPad. Prism (Version 5.01, San Diego, CA, USA), and the results were expressed by the mean standard error (SEM). The significant difference was presented in a *p* < 0.05, *p* < 0.01, and *p* < 0.001 format.

## 3. Results and Discussion

### 3.1. Effect of IND on Cell Viability of Coelomocytes

**Figure 1:**
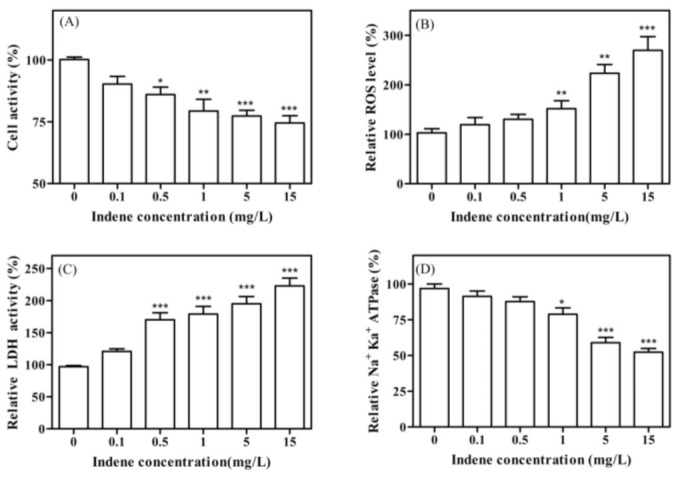
Relative cell viability (**A**), ROS (**B**), LDH (**C**) and Na^+^ K^+^ ATPase activity (**D**) after 24 h IND exposure. Values are means ± SEM (*n* = 3). Differences are considered statistically significant at *p* < 0.05, *p* < 0.01 and *p* < 0.001 and labeled with * and ** and ***, respectively.

Cell viability is a key parameter for assessing the degree of cytotoxicity induced by any exogenous substance [23,24]. We studied the changes in cell viability of earthworm coelomocytes 24 h after exposure to IND. As shown in Figure 1A, as the dosage of IND increased, cell viability showed a trend of gradual decline. When the IND concentration reached 15 mg/L, the cell viability decreased to 74.5% of the blank control group, indicating that IND inhibited cell viability. We further investigated the oxidative stress effect and mechanism of IND in coelomocytes due to oxidative stress, which can lead to cytotoxicity.

### 3.2. Changes in Intracellular Oxidative Stress Index Induced by IND

#### 3.2.1. Changes in ROS Level

As mentioned above, the toxicity of IND is likely to be caused by oxidative stress. In order to determine the mechanism by which IND induces intracellular oxidative stress, intracellular ROS levels were measured on a fluorometer after 24 h of IND exposure. Intracellular lactase can hydrolyze DCFH-DA infiltrating cells to form the DCFH carboxylate anion and retain it in cells. ROS can oxidize the DCFH carboxylate anion to form 2,7-dichlorofluorescein with detectable fluorescence [25,26].

As shown in Figure 1B, the ROS level in coelomocytes was low when the IND concentration was lower than 0.5 mg/L, and significantly increased when the IND concentration was greater than 0.5 mg/L. When the concentration was 15 mg/L, the intracellular ROS level reached 269.6% of the blank control group. Compared with the control group, after IND exposure, the intracellular redox state of coelomocytes was unbalanced and a large amount of ROS was produced, which may cause a series of oxidative damage. The continuous increase of ROS levels could lead to intracellular environmental oxidation, thus inducing oxidative stress. ROS plays a major role in normal cell signaling, and dysregulation of ROS can alter the expression of genes activated by redox mechanisms [27]. Excess ROS in cells may cause mitochondrial dysfunction [28], induce lipid peroxidation [29] and DNA strand breaks [30], and ultimately lead to cell death. Therefore, the activity changes of antioxidant enzymes were measured next.

#### 3.2.2. Changes of SOD and CAT Activity in Coelomocytes

SOD and CAT are very important antioxidant enzymes, which play a key role in maintaining redox homeostasis. SOD is the first line of defense against anti-oxidative damage, which can dismutate the ·O_2_^−^ in the body to generate H_2_O_2_ and O_2_, and the excess hydrogen peroxide generated will be cleared by CAT to generate oxygen molecules and water molecules [31].

**Figure 2:**
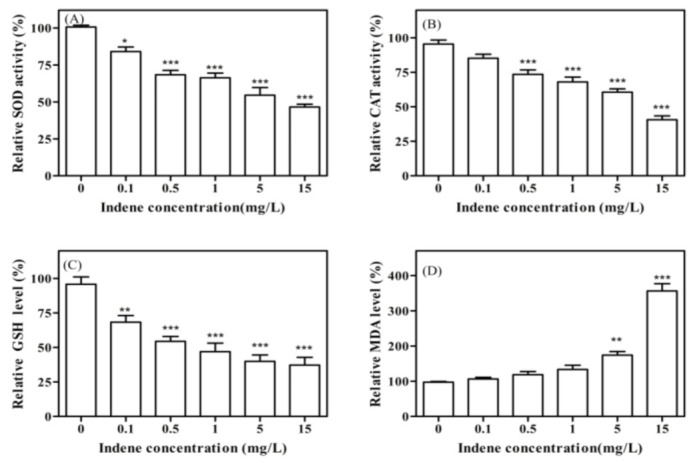
Activity changes of antioxidant enzymes and MDA level in coelomocyte after 24 h IND exposure. (**A**) SOD, (**B**) CAT, (**C**) GSH, (**D**) MDA level. Values are means ± SEM (*n* = 3). Differences are considered statistically significant at *p* < 0.05, *p* < 0.01 and *p* < 0.001 and labeled with * and ** and ***, respectively.

As shown in Figure 2A, with the increase in IND exposure concentration, the enzyme activity of SOD showed a trend of gradual decline. When the exposure concentration reached 15 mg/L, the enzyme activity decreased to 46.5% of the blank control group, which was due to the excessive ROS produced by coelomocytes exposed to IND, which led to partial SOD oxidation and thus reduced the total SOD activity. As shown in Figure 2B, with the increase in IND exposure concentration, the enzyme activity of CAT also showed a gradual downward trend. When the exposure concentration reached 15 mg/L, the enzyme activity decreased to 40.6% of the blank control group. Due to the fact that CAT could not eliminate the excessive hydrogen peroxide produced in coelomocytes, which exceeded the processing capacity of CAT, the enzyme activity of CAT was inhibited, which means that the antioxidant defense of CAT is reduced. It is concluded that exposure to IND disrupts the redox equilibrium in the coelomocytes of earthworms.

#### 3.2.3. Changes of GSH Content in Coelomocytes

In addition to antioxidant enzymes, the antioxidant defense system in the body also includes a variety of non-enzymes, including those that reduce glutathione pyruvate, as well as flavonoids and carotenoids such as GSH-Px, which catalyzes the H_2_O_2_ and GSH reaction, generating H_2_O and oxidizing GSH to GSSG. GSH has an antioxidant effect in vivo and is easy to be dehydrogenated by oxidation. One of its main functions is to eliminate ROS and regulate the redox balance in the body or cells.

As shown in Figure 2C, with the continuous increase in IND exposure concentration, the content of GSH in cells decreased gradually and finally decreased to 37.2% of the blank control group. This was because intracellular SOD and CAT were insufficient to resist excessive reactive oxygen species produced by coelomocytes, and the non-enzymatic antioxidant defense system in the body continued to deplete GSH in response to the oxidative stress effect in the body. Therefore, the content of GSH decreased.

### 3.3. Cell Membrane Damage

#### 3.3.1. Changes of MDA Level in Coelomocytes

The excessive accumulation of ROS in the body will trigger lipid peroxidation when attacking unsaturated fatty acids in cell membranes, resulting in oxidative damage and the formation of lipid peroxidation products such as MDA [32,33]. MDA is one of the most important products of membrane lipid peroxidation, which can also lead to the cross-linking polymerization of biological macromolecules such as proteins and nucleic acids. The content of MDA is often used as an indicator of lipid peroxidation. Therefore, the degree of oxidative damage to cells can be understood through the content of MDA [28].

As shown in Figure 2D, the MDA level in coelomocytes remained low after treatment with a lower IND concentration. However, after exposure to high concentrations of IND, MDA levels increased significantly with dose, reaching a maximum of 356.1% of the blank control group in a dose-dependent relationship compared with the control group. Our results suggest that high doses of IND exposure may lead to more MDA in the coelomocytes of earthworms and enhance oxidative damage to the coelomocytes.

#### 3.3.2. Changes of LDH Activity in Coelomocytes

As an important barrier between the cell and the external environment, the cell membrane can prevent extracellular substances from entering the cell and plays an important role in maintaining the stability and basic metabolism of the cell [34]. LDH is one of the biochemical indicators of cell membrane integrity. In healthy cells, LDH mainly exists in the cytoplasm. Once LDH is released extracellularly, it indicates increased osmosis of the plasma membrane or destruction of the cell membrane [35]. LDH release from the cell into culture is an indicator of irreversible cell death due to severe cell membrane damage.

As shown in Figure 1C, with the continuous increase in IND exposure concentration, the intracellular LDH activity increased significantly. When the IND exposure concentration was 15 mg/L, the intracellular LDH activity was 222.8% of the blank control group, indicating that IND damaged the cell membrane of the earthworm and increased the permeability of the cell membrane, resulting in more LDH being released from the cytoplasm.

#### 3.3.3. Changes of Na^+^ K^+^ ATPase Activity in Coelomocytes

ATPase is a membrane-bound enzyme that can catalyze the hydrolysis of ATP to ADP and phosphate radicals. The main role of ATPase is to transport cations through the cell membrane [36]. ATPase plays an important role in cells and is considered a sensitive index to determine toxicity [37]. Na^+^ K^+^ ATPase activity was positively correlated with membrane lipid fluidity, especially membrane fluidity, and membrane deformability.

As shown in Figure 1D, there was a significant dose reduction in Na^+^ K^+^ ATPase activity at 24 h after IND exposure. When the IND concentration reached 15 mg/L, Na^+^ K^+^ ATPase activity was significantly inhibited, decreasing to 52.33% of the blank control group. The results indicated that the ATPase system was damaged by IND, which interfered with the movement of Na^+^ K^+^ on the ion pump. This may lead to uneven entry of Na^+^ along the concentration gradient and uneven entry of water molecules along the osmotic gradient, which may lead to cell swelling and ultimately membrane rupture [38]. These results were consistent with the experimental results of LDH activity.

### 3.4. Molecular Mechanism of IND Interaction with SOD

#### 3.4.1. Changes in SOD Enzyme Activity In Vitro

**Figure 3:**
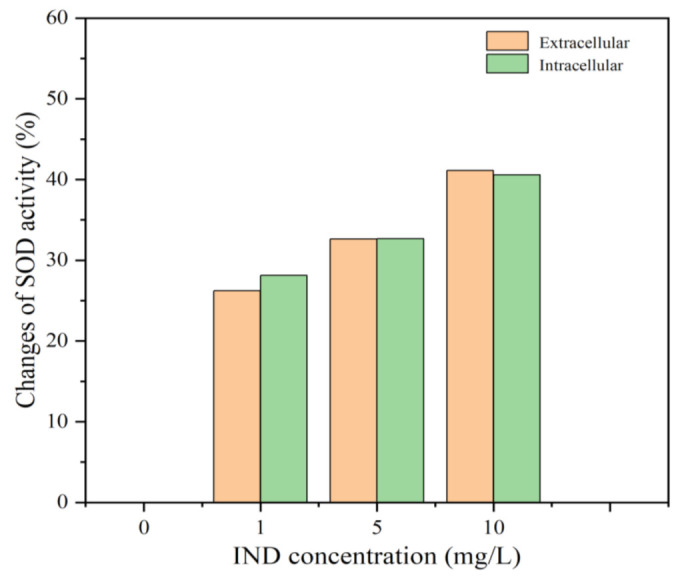
Contrast diagram of changes in SOD activity between intracellular and extracellular. Experimental Conditions: T = 298 K, pH = 7.4, c (SOD) = 1 μM, c (IND) = 0, 1, 5, 10 mg/L.

Because there is a specific correspondence between protein structure and physiological function, any structural change in a protein may lead to the loss of physiological function [39]. At the molecular level, the direct effect of IND on SOD enzyme activity was first measured. As shown in Figure 3, along with the increase in IND concentration, the change in SOD activity also showed an upward trend. This is because the change in enzyme structure may lead to changes in its activity and function. At the same time, the changes in intracellular and extracellular SOD activity in the body cavity were roughly the same, indicating that IND had the same intracellular and extracellular toxic effects on SOD activity. Therefore, in order to further explore the cause of the effect of IND on SOD activity, the binding modes of IND and SOD were studied, and the influence of the interaction between IND and SOD on the structure of the enzyme was analyzed.

#### 3.4.2. Study on Binding Constants, Thermodynamic Parameters and Acting Forces

The thermodynamic parameters of IND-SOD binding were calculated by detecting the change in heat during the process of IND-SOD binding by ITC technology [40,41]. Van der Waals forces, hydrogen bonds, hydrophobicity, and electrostatic forces are the four non-covalent bonding forces between proteins and small molecules [42]. The binding model can be simply judged according to thermodynamic parameters [43,44]: When ΔH^θ^ < 0 and ΔS^θ^ > 0, the main force is electrostatic force; When ΔH^θ^ < 0 and ΔS^θ^ < 0, the main force is Van der Waals force and hydrogen bond. When ΔH^θ^ > 0 and ΔS^θ^ > 0, the main force is the hydrophobic force.

**Figure 4:**
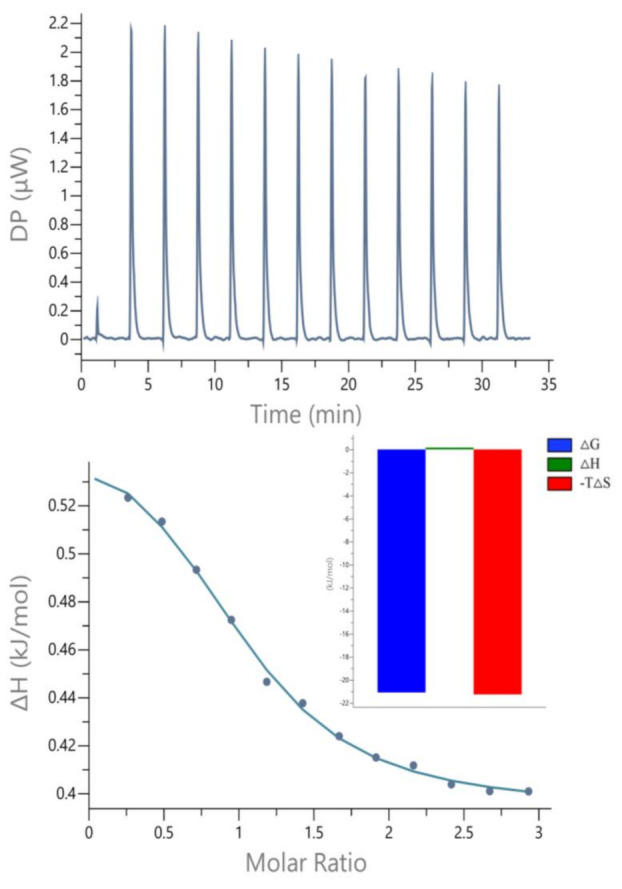
ITC profiles of the interaction between IND with SOD. Experimental conditions: c (SOD) = 1 mM, c (IND) = 10 mM, T = 298 K, pH = 7.4.

The ITC results are shown in Figure 4. The above diagram showed the raw data of heat (μcal/s) generated per drop relative to the titration time (minutes), and the below diagram showed the integral heat per drop adjusted for the molar ratio of dilution heat (kcal/mol) relative to IND/SOD. From the curve fitted by the software, the values of the enthalpy change ΔH, the binding constant K and the binding ratio N during the bonding process can be directly obtained (Table 1). When IND was titrated to a SOD solution, the peak values were all positive, indicating that their interactions were endothermic (ΔH > 0). A negative value of ΔG indicated that the binding process was spontaneous [45]. ΔH > 0 and ΔS > 0 indicating that the main force of IND and SOD binding was hydrophobic force. The value of ΔH was much less than 60 kcal m^−1^, indicating that the interaction between IND and SOD was non-covalent [46]. When the ligand-protein interaction was strong, the binding constant (K) was usually between 10^7^ and 10^8^ [47,48]. The order of magnitude of K_IND-SOD_ was 10^3^, indicating that the binding affinity between IND and SOD was relatively weak.

#### 3.4.3. Effect of IND on SOD Endogenous Fluorescence

Most proteins emit strong endogenous fluorescence due to the presence of tryptophan (Trp), tyrosine (Tyr), and phenylalanine (Phe) residues [49,50]. Preliminary experiments were carried out to test whether IFE could be ignored due to the possible presence of IFE caused by the absorption of ligands during excitation and emission radiation. The pre-experimental results show that under the condition of 290–450 nm, the absorbance of the experimental system is greater than 0.05, resulting in an internal filtration effect of more than 5%, so the internal filtration effect of the experimental system cannot be ignored [51,52].

**Figure 5:**
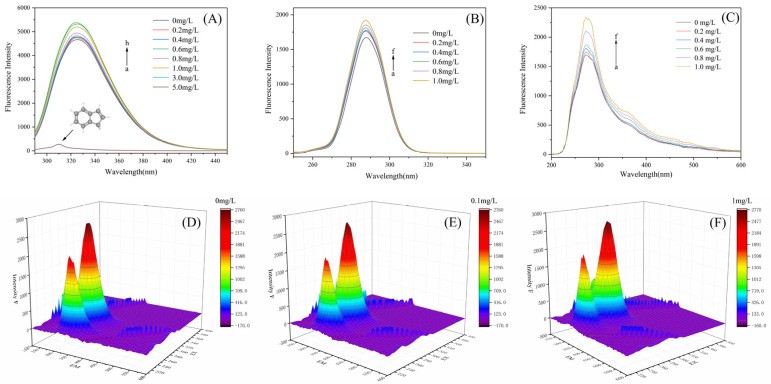
Fluorescence spectra of SOD-IND system. (**A**) Fluorescence spectra; (**B**) Synchronous fluorescence spectrum (Δλ = 15 nm); (**C**) RLS; (**D**–**F**) EEM Experimental Conditions: T = 298 K, pH = 7.4, λex = 280 nm, c (SOD) = 5 μM, c (IND) of (**A**) a–h: 0, 0.2, 0.4, 0.6, 0.8, 1.0, 3.0, 5.0 mg/L; c (IND) of (**B**) a–f: 0, 0.2, 0.4, 0.6, 0.8, 1.0 mg/L; c (IND) of (**C**) a–f: 0, 0.2, 0.4, 0.6, 0.8, 1.0 mg/L.

The fluorescence light emission spectrum of SOD with internal filtration is shown in Figure 5A. With the increase in IND concentration, the fluorescence intensity of SOD gradually increases, but the shape and position of the fluorescence emission peak remain unchanged. IND generated a fluorescence sensitization effect on SOD, indicating that IND interacted with the chromophore of SOD and that SOD and IND form a micelle group, which explained this phenomenon. The chromophore was surrounded by micelles, which reduced the energy loss caused by collision, improved the fluorescence quantum yield, and eventually led to the fluorescence sensitization of the IND-SOD system [53,54]. In addition, the decrease of polarity near the chromophore also led to an increase in fluorescence intensity [55,56].

Since most of the fluorescence of SOD came from Tyr residue, the fluorescence intensity of Tyr was detected by using the synchronous fluorescence spectrum [57]. When Δλ was fixed at 15 nm, the synchronous fluorescence spectrum could reflect the information of the Tyr residue microenvironment [58,59].

It can be seen from Figure 5B that the variation trend of synchronous fluorescence intensity was consistent with the emission fluorescence spectrum in Figure 5A. Similarly, IND hardly changed the fluorescence signal of Tyr, and with the increase in IND concentration, the fluorescence intensity of Tyr in SOD gradually increased. These results indicated that SOD molecules unfold after interaction with IND, exposing more Tyr residues in the protein. Therefore, the local refractive index increased, which confirmed the interaction between IND and Tyr.

In the analysis of protein structure, EEM is an important method, because it can make the study of protein characteristics and conformational changes more scientific and reliable. Once the protein conformation changes, the excitation or emission wavelength around the fluorescence peak will shift, a new peak will appear, or the existing peak will disappear. As shown in Figure 5D–F, the conformational and microenvironmental changes of SOD were studied by comparing the spectral changes in the presence and absence of IND with the corresponding parameters in Table 2. Peak 1 and peak 2 are first-order rayleigh scattering peaks (λex = λem) and second-order rayleigh scattering peaks (λex = 2 λem), respectively [60,61]. After IND exposure, the signal values of these two peaks were significantly enhanced, indicating that IND and SOD formed a complex and thus enhanced the scattering effect. Peak 3 mainly shows the spectral characteristics of Trp and Tyr residues. The peak value increased slightly, indicating that IND had a fluorescence sensitization effect on SOD. Peak 4 mainly reflected the information of polypeptide skeleton changes caused by π-π* transition, and IND exposure enhanced the fluorescence value of this peak, indicating that the combination of IND and SOD would cause the polypeptide skeleton of SOD to slightly expand, resulting in conformational changes. All these phenomena showed that the combination of IND and SOD could cause the conformational and microenvironmental changes of SOD.

#### 3.4.4. Effect of IND on SOD Particle Size

The change of SOD structure and conformation may be affected by the change of protein particle size after IND binding [47,54]. Resonant light scattering (RLS) spectroscopy was used to study the particle size changes of the system before and after IND exposure. The intensity of RLS is proportional to the volume of the dispersed particles [62,63].

Figure 5C is the RLS spectra of the SOD-IND complex. As is shown in the figure, with the increase in IND concentration, the RLS strength of the system gradually increased, indicating that SOD attracted IND and aggregated through intermolecular force to form the SOD-IND complex with a higher absorption rate than SOD.

#### 3.4.5. Effects of IND on SOD Skeleton and Aromatic Amino Acid Microenvironment

UV–visible absorption spectroscopy is often employed to investigate the changes in the structure of proteins and the formation of protein–ligand complexes [64,65]. The UV-Vis absorption spectra of proteins usually have two peaks, located at 208 nm and 280 nm, respectively. The strong peak at 208 nm can reflect the characteristics of the protein skeleton, which is caused by π → π* electron transition in the C=O polypeptide chain skeleton [66,67]. The weak peak at 280 nm can reflect the change of chromophore microenvironment, which is caused by the absorption of light by aromatic amino acids [61,68].

**Figure 6:**
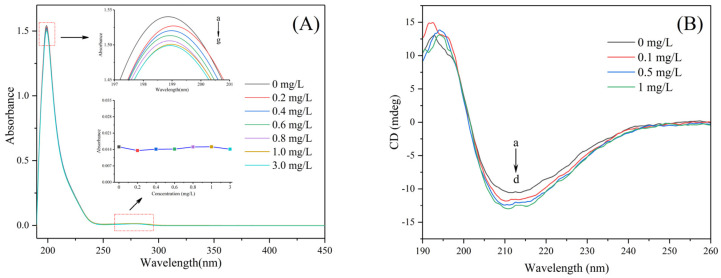
Changes of primary and secondary structure of SOD after IND exposure. Experimental Conditions: T = 298 K, pH = 7.4, c (SOD) of (**A**): 1 μM, c (SOD) of (**B**): 5 μM, c (IND) of (**A**) a–g: 0, 0.2, 0.4, 0.6, 0.8, 1.0, 3.0 mg/L, c (IND) of (**B**) a–d: 0, 0.1, 0.5, 1 mg/L.

As can be seen from Figure 6A, SOD had two main absorption peaks, which were located at about 205 nm and 280 nm, respectively. After exposure to IND, the skeleton peak of SOD protein decreased, but there was no obvious shift in the maximum absorption wavelength. The effect of IND on the main structure of the SOD peptide was obvious, but the effect on the microenvironment around the aromatic amino acid residues was small. This indicated that IND affected the peptide bond, destroyed the skeleton structure of SOD, and affected the spatial configuration of SOD.

#### 3.4.6. Effect of IND on the Secondary Structure of SOD

Circular dichroism (CD) can be used to detect the secondary structure changes of protein macromolecules. In this research, the effect of IND on SOD conformation was studied using CD [69,70]. In general, the far-ultraviolet CD spectra of proteins have two negative peaks around 208 nm and 222 nm, which represent the n → π* and π → π* electron transitions of the α-helix, respectively [71,72].

Figure 6B showed the CD spectra of SOD combined with IND at different concentrations. As shown in the figure, with the increase in IND concentration, the intensity of the SOD negative band near 208 nm gradually increased, indicating the changes in SOD secondary structure. CDPro software was further used to analyze the changes in the percentages of α helix, β folding, turning angle, and irregular curling in SOD. As shown in Table 3, SOD itself contains 20.8% α helix, 42.9% β folding, 14.2% β turning angle and 22.1% irregular curling. After the addition of IND, the content of α helix in SOD increased to 23.1% and the content of β fold in SOD decreased to 32.1%, resulting in protein misfolding and partial degeneration. These results showed that IND had an interaction with the main chain of SOD, which made the protein become loose, broke the hydrogen bond of SOD, and acted with amino acid residues, resulting in partial denaturation and a conformational change of SOD [73,74]. These structural changes make the SOD structure more expanded, the active center enzyme more exposed, and thus easier to combine with the substrate, thus improving the enzyme activity [28].

#### 3.4.7. Molecular Docking of IND and SOD Binding Sites and Binding Models

In order to better understand the interaction between SOD and IND and elucidate the internal mechanism of IND induced changes in SOD, MOE was used to simulate the specific binding sites of the IND and SOD [75]. This technique can find the preferred binding sites of ligands on specific proteins and confirm the experimental results of spectra.

**Figure 7:**
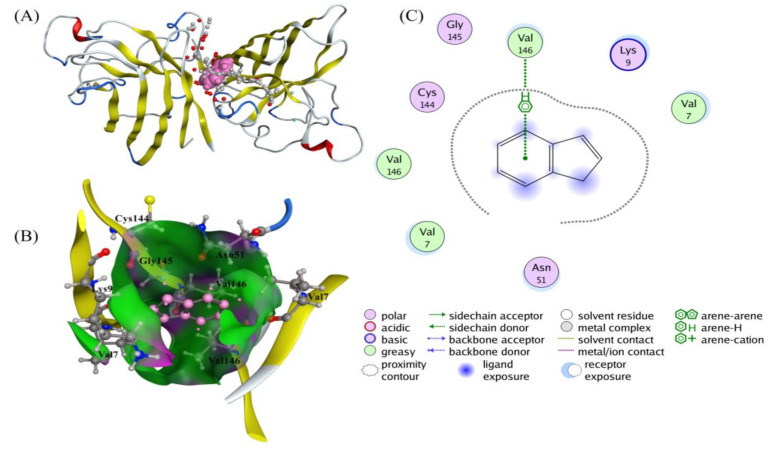
IND and SOD docking configuration files. (**A**) SOD is represented by cartoon, IND is represented by ball and stick, and active site is represented by filling mode. (**B**) Gaussian contact pattern superposed with ligand and receptor: pink for hydrogen bonding, green for hydrophobic preference, blue for mild polarity. IND is represented by sticks, and related amino acid residues are represented by balls and sticks. (**C**) Ligand interactions between IND and related amino acid residues.

The software calculation showed that the minimum binding free energy of the SOD-IND system is -5.6499 kJ /mol under the condition of 298 K. As can be seen from Figure 7A, SOD had two subunits, each of which contained a Cu atom and a Zn atom. Electrostatic force and hydrophobic force connect the two subunits together [76]. Figure 7A showed that IND was bound to the interface of the two subunits preferentially after molecular docking. Figure 7B showed that the binding sites of IND to SOD included Val 7, Lys 9, Asn 51, Cys 144, Gly 145, and Val 146 of one subunit and Val 7 and Val 146 of the other subunit. Figure 7C showed that IND and SOD were connected through an arene-H bond. The distance was 4.53 Å, and the bond energy was −1.5 kJ/mol. Molecular simulation results showed that the binding sites of IND and SOD were far from the active center of the enzyme, so IND could not directly reach the active center of the enzyme. However, IND may increase the pocket near the active center of the enzyme molecule by changing the structure of SOD, so that SOD could contact with the substrate more fully, leading to an increase in the activity of enzyme.

The solvent accessible surface area (SASA) of SOD is 16,218.43 Å^2^, and that of SOD-IND is 16,686.33 Å^2^, which indicates that the addition of IND causes the disfolding of the SOD protein structure, thus exposing the hydrophobic groups buried inside the molecule. This also confirmed the experimental result that the hydrophobic force played a dominant role in the interaction between IND and SOD.

## 4. Conclusions

Based on the background of environmental toxicology and modern instrumental analysis science, this research studied the oxidative stress induced by IND, its related toxic effects, and specific toxic mechanisms at the cellular and molecular levels.

**Figure 8:**
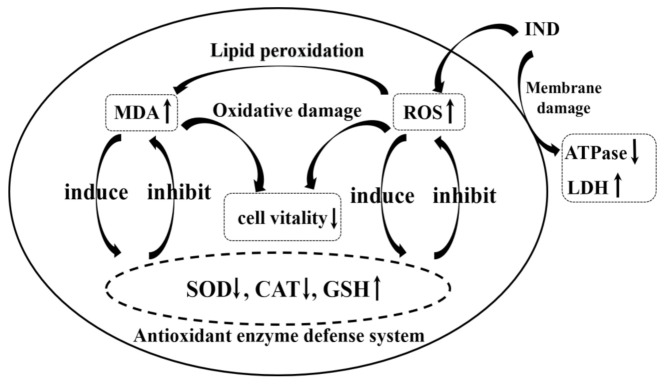
The diagram of the relationship of oxidative stress indexes. The arrow represents the effect of the former on the latter.

The cytotoxicity mechanism of IND is shown in Figure 8. The intracellular oxidative stress indexes mainly included cell vitality, ROS level, antioxidant enzyme (SOD, CAT) activity, GSH content, and cell membrane damage. The experimental results showed that IND exposure resulted in a large amount of intracellular ROS accumulation, an imbalance of intracellular redox balance, and an oxidative stress effect in coelal cells. In order to eliminate excess ROS, the activities of the enzymatic antioxidants SOD and CAT were inhibited. When SOD and CAT were insufficient to resist oxidative stress, the non-enzymatic antioxidant GSH was continuously consumed. In terms of cell membrane damage, excess ROS induced lipid peroxidation of the cell membrane and significantly increased intracellular MDA levels. At the same time, as the activities of LDH and Na^+^ K^+^ ATPase on the cell membrane were affected, the level of extracellular LDH was increased and the activity of Na^+^ K^+^ ATPase was decreased significantly, resulting in cell swelling and increased cell membrane permeability. Finally, cell membranes ruptured, and cell vitality decreased to 74.5% of the control group. In addition, biophysical methods such as multispectral, ITC and molecular docking studies were used in this research to deeply understand the molecular mechanism of IND induced SOD activity increase. The results showed that IND induced the fluorescence sensitization of SOD and changed the microenvironment of Tyr residue. The binding of IND to SOD was mainly through the hydrophobic force, which led to the loosening of the polypeptide chain and the expansion of the secondary structure of SOD. The results of molecular docking showed that IND was preferentially bound to the interface of two SOD subunits and was bound to SOD by an arene-H bond, thus affecting the change in SOD enzyme activity.

In this research, a combination of cellular and molecular methods was used to evaluate the pollution risk of IND to the environment and human health, providing a basis for the pollution risk assessment of IND to the environment and human health.

## Figures and Tables

**Table 1:** Binding parameters of the interaction between SOD and IND.

T (K)	K	N	ΔH (kJ M^−1^)	ΔG (kJ M^−1^)	ΔS (kJ M^−1^k^−1^)
298.15	4.95 × 10^3^	0.999	0.166	−21.1	0.0714

**Table 2:** Change information of SOD EEM fluorescence peak after combined with IND.

IND (mg/L)	Peak 3		Peak 4	
	Peak Position λex/λem (nm/nm)	Intensity	Peak Position λex/λem (nm/nm)	Intensity
0	280/325	2752.76	230/330	1499.97
0.1	280/325	2757.76	230/325	1492.85
1	280/325	2760.76	230/320	1396.85

**Table 3:** Secondary structure content of SOD before and after exposure to IND.

IND Concentration (mg/L)	Secondary Structural Content in Enzyme (%)			
	α-Helix	β-Sheet	Turn	Unordered
0	20.8	42.9	14.2	22.1
0.1	21.0	41.4	15.6	22.0
0.5	22.3	35.6	18.2	23.9
1	23.1	32.1	20.2	24.6

## Data Availability

Not applicable.

## Supplementary Materials

The following supporting information can be downloaded at: https://www.mdpi.com/article/10.3390/toxics11020136/s1.
