# Supplementary material for: Cytotoxicity and Oxidative Stress Effects of Indene on Coelomocytes of Earthworm (Eisenia foetida): Combined Analysis at Cellular and Molecular Levels
Source: Toxics. 2023 Jan 30;11(2):136. doi: 10.3390/toxics11020136 (PMC9961689; doi:10.3390/toxics11020136)
Supplement: Supplementary file 1 [file toxics-11-00136-s001.zip › toxics-2135637-supplementary.pdf]

2.3.1 Extraction of earthworm coelomocytes and IND exposure experiment

The intestinae were cleared for 24 h in a disposable petri dish containing wet filter paper under dark conditions. The earthworm coelomocytes were extracted from a mixture of 5% ethanol anhydrous, 95% normal saline, 2.5 mg·mL-1 Na2EDTA and 10 mg·mL-1 guaifenesin. We centrifuged the earthworm coelomocyte fluid (3500 RPM, 5 min), poured out the supernatant and washed it twice with normal saline under the same centrifugation conditions. The coelomocytes were added to the prepared cell medium (RPMI 1640 medium: Foetal Bovine Serum : mixture of cylinamycin =89:10:1), and beaten evenly. The coelomocyte fluid was then diluted to the desired concentration.

2.4.4 Fluorescence spectrum experiment

Fluorescence spectrum and synchronous fluorescence spectrum were measured on a fluorescence spectrophotometer. The photomultiplier voltage was set as 700V, the sampling interval was 0.2 nm, the scanning speed was 1200 nm/min, the width of excitation and emission slit was 5.0 nm, the excitation wavelength was 280 nm, and the scanning range of emission spectrum was 290 - 450 nm. Synchronous 15 excitation wavelength 265 nm, scanning range 250 - 350 nm, $\Delta\lambda = 15$ nm.

Excitation-Emission-Matrix (EEM) was measured on a fluorescence spectrophotometer. The photomultiplier voltage was set as 650 V, the width of excitation and emission slit was set as 5.0 nm, and the scanning speed was set as 1200 nm/min. The scanning interval was set as follows: Ex = 200 - 400 nm, Em = 220 - 600 nm.

Resonant light scattering spectrum (RLS) was measured on a fluorescence spectrophotometer. The photomultiplier voltage was set as 650 V, the sampling interval was 0.2 nm, the scanning speed was 1200 nm/min, the width of excitation and emission slit was 5.0 nm, the excitation wavelength was equal to the emission wavelength, and the scanning range was 200 - 600 nm.
